# In Vitro Activitiy of Rezafungin in Comparison with Anidulafungin and Caspofungin against Invasive Fungal Isolates (2017 to 2022) in China

**DOI:** 10.3390/jof10060397

**Published:** 2024-05-31

**Authors:** Simin Yang, Feifei Wan, Min Zhang, Huiping Lin, Liang Hu, Ziyi Zhou, Dongjiang Wang, Aiping Zhou, Lijun Ni, Jian Guo, Wenjuan Wu

**Affiliations:** Department of Laboratory Medicine, Shanghai East Hospital, Tongji University School of Medicine, 1800 Yuntai Road, Pudong New District, Shanghai 200123, China; yang_kisa@hotmail.com (S.Y.); wan_xmu@163.com (F.W.); zhangmin951010@163.com (M.Z.); linhuiping2021@126.com (H.L.); hu740646282@sina.com (L.H.); m13031332829@163.com (Z.Z.); wdj_2011@sina.com (D.W.); 17721329309@126.com (A.Z.); ni1031@126.com (L.N.)

**Keywords:** echinocandins, rezafungin, anidulafungin, caspofungin, invasive fungal infection

## Abstract

The efficacy of different echinocandins is assessed by evaluating the in vitro activity of a novel antifungal, rezafungin, against invasive fungal isolates in comparison with anidulafungin and caspofungin. Using the broth microdilution (BMD) method, the susceptibility of 1000 clinical *Candida* isolates (including 400 *C. albicans*, 200 *C. glabrata*, 200 *C. parapsilosis*, 150 *C. tropicalis* and 50 *C. krusei*) and 150 *Aspergillus* isolates (100 *A. fumigatus* and 50 *A. flavus*) from the Eastern China Invasive Fungi Infection Group (ECIFIG) was tested for the antifungals including anidulafungin, rezafungin, caspofungin and fluconazole. The echinocandins showed strong activity against *C. albicans* that was maintained against fluconazole-resistant isolates. The GM MIC (geometric mean minimum inhibitory concentration) value of rezafungin was found to be comparable to that of anidulafungin or caspofungin against the five tested common *Candida* species. *C. tropicalis* exhibited higher resistance rates (about 8.67–40.67% in different antifungals) than the other four *Candida* species. Through the sequencing of *FKS* genes, we searched for mutations in echinocandin-resistant *C. tropicalis* isolates and found that all displayed alterations in *FKS1* S654P. The determined MEC (minimal effective concentration) values against *A. fumigatus* and *A. flavus* for rezafungin (0.116 μg/mL, 0.110 μg/mL) are comparable to those of caspofungin (0.122 μg/mL, 0.142 μg/mL) but higher than for anidulafungin (0.064 μg/mL, 0.059 μg/mL). Thus, the in vitro activity of rezafungin appears comparable to anidulafungin and caspofungin against most common *Candida* and *Aspergillus* species. Rezafungin showed higher susceptibility rates against *C. glabrata*. Rezafungin indicates its potent activity for potential clinical application.

## 1. Introduction

*Candida* and *Aspergillus* species are the most important and common pathogens causing invasive fungal infections, mostly in immunocompromised patients. The incidence of invasive candidiasis (IC) and invasive aspergillosis (IA) has been increasing in recent years and is associated with high morbidity, mortality and clinical costs [1]. Besides the most prevalent pathogen *Candida albicans*, the incidence of infections linked with four other common non-*albicans Candida* species has risen in the last few decades, namely for *Candida glabrata*, *Candida parapsilosis*, *Candida tropicalis* and *Candida krusei* [2,3]. To avoid life-threatening consequences, effective antifungals should be promptly administered to patients with IC and IA [4]. Data from the Global Antifungal Surveillance Group show that the rates of *C. tropicalis* resistance to fluconazole (6.5%) and voriconazole (8.4%) were higher in the Asia–Pacific than in other regions during 1997–2007 [5]. The China Hospital Invasive Fungal Surveillance Net (CHIF-NET) revealed that from 2014 to 2018, the resistance rates of *Candida tropicalis* to fluconazole and voriconazole increased from 5.7% to 21.0% and from 5.7% to 21.4%, respectively [6]. The impact of fungal infections has been exacerbated by the rise in antifungal resistance [7].

With the frequent use of antifungals, drug resistance has been increasing gradually, resulting in the limitation of the available, effective antifungals [8]. Therefore, the development of antifungal agents with a broad antibacterial spectrum, convenient use and robust safety has important clinical significance and extensive market prospects. Azoles were previously the most commonly used antifungals, as recommended in the candidiasis and aspergillosis guidelines. However, drug–drug interactions are prominent and can occur at several stages of azole metabolism. Almost all azoles interact with cytochrome P450 enzymes [9]. In recent years, echinocandins have been included in the therapy guidelines and are highly recommended due to their high antifungal efficacy and lower incidence of adverse reactions [10,11,12]. The results of the SENTRY Antifungal Surveillance Program in 2013 showed that rates of resistance ranged from 0.0% to 2.8% for echinocandin in *Candida* species and were 11.9% and 11.6% for fluconazole against *C. glabrata* and *C. tropicalis*, respectively [13]. With the increasing use of echinocandin, there has been a decrease in the echinocandin susceptibility of *Candida* species together with gradually increasing cases of clinical treatment failure, which has attracted extensive attention [14]. From 2001 to 2010, the echinocandin resistance rate of *C. glabrata* in Duke Hospital increased from 4.9% to 12.3% [15].

Common echinocandins include caspofungin, micafungin, anidulafungin and rezafungin. In China, caspofungin and micafungin have been approved for use by the National Medical Products Administration (NMPA), but anidulafungin and rezafungin are still undergoing clinical trials. Rezafungin is a novel echinocandin that requires only once-weekly dosing for the treatment of IC. Results from the Phase 2STRIVE trial showed that rezafungin has a prolonged pharmacokinetic activity in comparison with anidulafungin and caspofungin [16]. Phase 3 clinical data demonstrated that rezafungin is non-inferior to caspofungin, which further supported its safety and efficacy [17]. Anidulafungin and rezafungin were approved for clinical use by the US Food and Drug Administration (FDA) in 2006 and 2023, respectively [18,19]. The use of echinocandins in antifungal therapy is expected to become more common, necessitating the clinical evaluation of the echinocandins.

In this study, we compared the novel antifungal rezafungin with anidulafungin and caspofungin based on their activity against five common *Candida* species and two common *Aspergillus* species. In vitro antifungal susceptibility testing was used to assess the efficacy of these different echinocandins.

## 2. Materials and Methods

### 2.1. Antifungal Agents

Anidulafungin (range 0.015–16 mg/L), rezafungin (range 0.015–16 mg/L) and caspofungin (range 0.015–16 mg/L) powders were purchased from MedChemExpress (Sollentuna, Sweden). Fluconazole (range 0.25–256 mg/L) powder was purchased from the National Institutes for Food and Drug Control (Beijing, China). Drug stock solutions of these echinocandin compounds were prepared with an initial concentration of 32 μg/mL and used for double gradient dilution in 96-well untreated polystyrene plates. The testing of anidulafungin, rezafungin and caspofungin was all carried out using two duplicates according to the Clinical and Laboratory Standards Institute (CLSI) M27 4th edition and M38 3rd edition [20,21]. The negative control well contained only the RPMI1640 medium and no antifungal medium.

### 2.2. Strains

A total of 1000 clinical *Candida* isolates and 150 clinical *Aspergillus* isolates were tested in this study, including 400 *C. albicans*, 200 *C. glabrata*, 200 *C. parapsilosis*, 150 *C. tropicalis*, 50 *C. krusei,* 100 *A. fumigatus* and 50 *A. flavus* isolates. The isolates were all from the Eastern China Invasive Fungi Infection Group (ECIFIG) and had been collected from different tertiary hospitals in eastern China between January 2017 and December 2022. *C. parapsilosis* (ATCC 22019), *C. krusei* (ATCC 6258) and *A. fumigatus* (ATCC MYA-3626) were used as quality control (QC) strains for each batch of the experimental procedure in this study. Ethical approval was obtained from Shanghai East Hospital (Tongji University School of Medicine) on 4 July 2021. The approval No. is [2021] (061).

### 2.3. Identification of Isolates

After acquisition, all isolates were identified to species level using a combination of morphological analysis and matrix-assisted laser desorption/ionization-time of flight mass spectrometry (MALDI-TOF MS EXS3000, Zybio, China) with reference to database version V1.0.20.2 and then stored at −80 °C. If the MALDI-TOF results were unreliable(score < 2), sequencing of the internal transcribed spacer (ITS) regions of ribosomal DNA (ITS1/ITS4) was conducted for identification.

### 2.4. Antifungal Susceptibility Testing

The strains were subcultured onto Sabouraud dextrose agar culture medium (Oxoid, UK) at least twice to ensure their purity and activity prior to incubation at 35 °C for 24 h. Antifungal susceptibility testing of the *Candida* and *Aspergillus* isolates was performed by broth microdilution (BMD) using anidulafungin, rezafungin, caspofungin and fluconazole (for comparison) in accordance with the CLSI reference standard [20,21]. The MIC (minimum inhibitory concentration) values for anidulafungin, rezafungin and caspofungin were determined after 24 h of incubation at the lowest concentration of the drug that achieved 50% growth inhibition compared to the control well [22,23]. The MECs (minimal effective concentration) were read as the lowest concentration of antifungals that led to the growth of small, rounded, compact hyphal forms [24].

### 2.5. FKS Mutation Sequencing

Sanger sequencing was used to sequence the internal transcribed spacer regions ITS1 and ITS2 from 13 *C. tropicalis* isolates displaying MIC values corresponding to echinocandins resistance. Sequences of *FKS* genes were compared to the relevant reference sequences.

### 2.6. Data Analysis

All data were statistically analyzed using IBM SPSS Statistics 23 and GraphPad Prism 9, and results are expressed as the MIC/MEC range, MIC/MEC_50_, MIC/MEC_90_ and GM (geometric mean) MIC/MEC. Isolates were classified as susceptible, intermediate or resistant to anidulafungin and caspofungin.

## 3. Results

**Figure 1:**
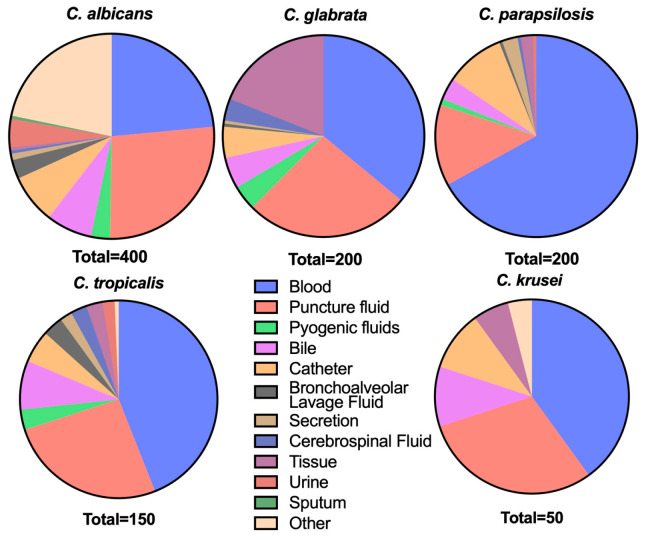
The source distribution of the five *Candida* species.

A total of 1000 clinical *Candida* isolates (*C. albicans*, n = 400; *C. glabrata*, n = 200; *C. parapsilosis*, n = 200; *C. tropicalis*, n = 150; *C. krusei*, n = 50) and 150 *Aspergillus* isolates (*A. fumigatus*, n = 100; *A. flavus*, n = 50) were applied in assessing the antifungals of anidulafungin, rezafungin, caspofungin and fluconazole using BMD. More than half of the isolates were collected from blood and puncture fluid. The source distribution of the clinical isolates is shown in Figure 1. The overall performance of the three echinocandins against *Candida* isolates is shown in Table 1. The values determined for the quality control strains for each batch all fell within the acceptable ranges within twofold dilutions following the respective standard conditions (*C. parapsilosis* ATCC 22019, anidulafungin 1–2 mg/L, caspofungin 0.5–1 mg/L, rezafungin 1–2 mg/L, fluconazole 1–2 mg/L; *C. krusei* ATCC 6258, anidulafungin 0.125 mg/L, caspofungin 0.25–0.5 mg/L, rezafungin 0.125 mg/L, fluconazole 16 mg/L).

### 3.1. Candida albicans

**Figure 2:**
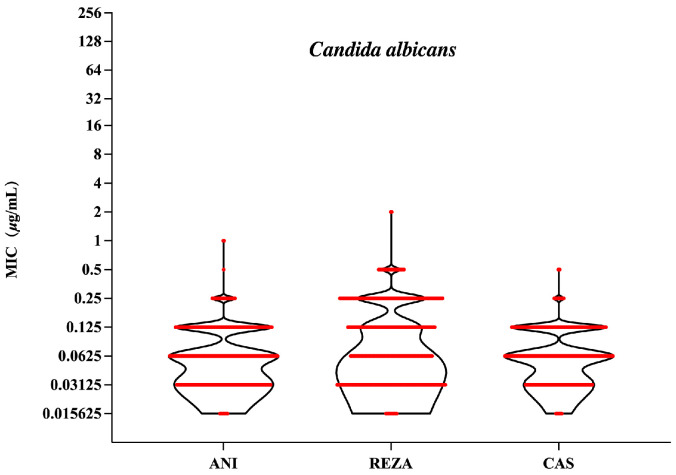
MICs of anidulafungin, rezafungin and caspofungin for *C. albicans* isolates. ANI, anidulafungin; REZA, rezafungin; CAS, caspofungin.

The GM MIC of rezafungin against *C. albicans* was 0.095 μg/mL, higher than that of anidulafungin (GM MIC = 0.068 μg/mL) and caspofungin (GM MIC = 0.069 μg/mL) (*p* < 0.001, Figure 2). Rezafungin (MIC_50/90_, 0.12/0.25 μg/mL) inhibited all *C. albicans* isolates at 2 μg/mL, with MIC_50/90_ values corresponding to one dilution gradient higher than anidulafungin and caspofungin. The susceptibility rates were 99.25% and 100% for anidulafungin and caspofungin, respectively (Table 1). Rezafungin exhibited comparable antifungal activity to anidulafungin and caspofungin against *C. albicans*, and this strong activity was maintained against fluconazole-resistant isolates.

### 3.2. Non-Albicans Candida

The MIC_50_ and MIC_90_ values of rezafungin determined against 200 clinical isolates of *C. glabrata* (ranged from 0.03 to 1 μg/mL), were 0.125 and 0.25 μg/mL, respectively, comparable to those of anidulafungin and caspofungin, toward which around 2% and 1.5% of the isolates were resistant (Table 1). The rate of susceptibility to rezafungin (99%) was higher than for the other four common *Candida* species.

The MIC_50/90_ values of rezafungin against *C. parapsilosis* were the same as those of anidulafungin but correspond to one to two dilution gradients higher than caspofungin. All three echinocandins demonstrated potent activity against *C. parapsilosis* and *C. krusei*. None of the 200 *C. parapsilosis* and 50 *C. krusei* isolates were found to be resistant to anidulafungin or caspofungin, and the rate of susceptibility to rezafungin was also very high (97.5% and 92%). Caspofungin showed more potent activity against *C. parapsilosis* with a GM MIC of 0.551 μg/mL, and anidulafungin exhibited its highest potency against *C. krusei* with both MIC_50_ and MIC_90_ values being 0.12 μg/mL (Table 1).

Of the five *Candida* species, the highest resistance rates were observed for *C. tropicalis* (8.67% for anidulafungin, caspofungin and rezafungin). Compared to the other echinocandins, rezafungin performed similarly against *C. tropicalis* and was more effective than fluconazole, for which the susceptibility rate was 91.33%.

**Figure 3:**
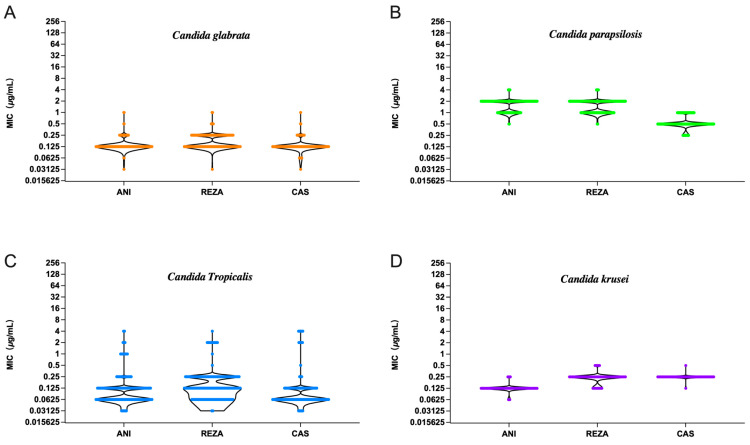
MICs of anidulafungin, rezafungin and caspofungin against four common non-*albicans Candida* isolates. (**A**): MICs against *C. glabrata*; (**B**): MICs against *C. parapsilosis*; (**C**): MICs against *C. tropicalis*; (**D**): MICs against *C. krusei*. ANI, anidulafungin; REZA, rezafungin; CAS, caspofungin.

The GM MIC values of rezafungin were 0.174, 1.564, 0.163 and 0.240 μg/mL against *C. glabrata*, *C. parapsilosis*, *C. tropicalis* and *C. krusei*, respectively. Rezafungin was comparable to anidulafungin and caspofungin in terms of activity against the tested *C. parapsilosis* and *C. krusei.* The rates of susceptibility for *C. glabrata* were higher for rezafungin than for both anidulafungin and caspofungin (Figure 3).

### 3.3. Activity of Fluconazole against Candida Species

Echinocandins showed potent activity against the tested *C. albicans,* and this activity was maintained against fluconazole-resistant isolates. For *C. albicans*, the rate of resistance was 6.75% against fluconazole and 0.5% and 0% against anidulafungin and caspofungin, respectively. The MIC_50_, MIC_90_ and GM MIC values of the echinocandins in fluconazole-sensitive *C. albicans* were comparable to those in fluconazole-intermediate/resistant *C. albicans* (Table 2). A much higher resistance rate was observed for fluconazole (40.67%) than the echinocandins when tested using *C. tropicalis*. *C. krusei* was assumed to be intrinsically resistant to fluconazole, whereas none of the isolates showed resistance to anidulafungin or caspofungin, and the rate of susceptibility to rezafungin was determined to be 92% given that rezafungin currently does not have “intermediate” or “resistant” criteria (Table 1).

### 3.4. Aspergillus Species

**Figure 4:**
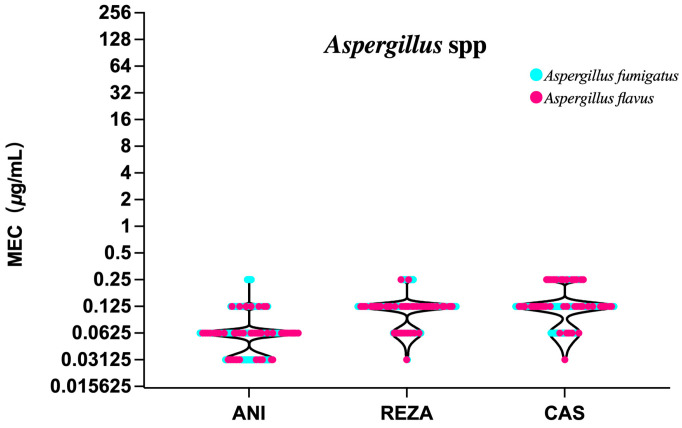
MECs of anidulafungin, rezafungin and caspofungin against two common *Aspergillus* isolates. ANI, anidulafungin; REZA, rezafungin; CAS, caspofungin.

All three echinocandins showed potent activity against *A. fumigatus* and *A. flavus*. None of the 100 *A. fumigatus* and 50 *A. flavus* isolates were NWT to anidulafungin or caspofungin. The GM MEC values for rezafungin were 0.116 and 0.110 μg/mL against *A. fumigatus* and *A. flavus*, respectively. Rezafungin therefore shows activity comparable to caspofungin but lower than anidulafungin against *Aspergillus* species (Table 3, Figure 4).

### 3.5. Resistance Mutations in Echinocandin-Resistant C. tropicalis Strains

On the basis of susceptibility patterns, we observed more echinocandin-resistant isolates from *C. tropicalis*. The rates of echinocandin resistance were higher for isolates of *C. tropicalis* than of other *Candida* species, with 8.67% of isolates resistant to anidulafungin and caspofungin. Given these high resistance rates, we analyzed the clinical characteristics of the 13 echinocandin-resistant clinical isolates and found that most (9/13) were isolated from patients in the same neurosurgery ward (Supplementary Table S1). We searched for resistance mutations by sequencing the *FKS* genes for all isolates with a MIC higher than clinical breakpoint (CBP) in *C. tropicalis* and found that all displayed mutations in *FKS1*. Alterations corresponding to the *FKS1* hot spot S654P were found in all 13 resistant isolates. One isolate had an S654P alteration plus R1220T in *FKS1*. One case of alterations in the *FKS1* hot spot S654 plus G324R was also found. Details of the *FKS1* mutations and the MICs of the echinocandin-resistant *C. tropicalis* are summarized in Table 4.

## 4. Discussion

*Candida* and *Aspergillus* species are the most common opportunistic fungal pathogens leading to invasive fungal infections with high morbidity and mortality, especially in healthcare and immunocompromised patients [25]. More than 90% of ICs are caused by the five common *Candida* species, namely *C. albicans*, *C. glabrata*, *C. parapsilosis*, *C. tropicalis* and *C. krusei*, each with distinct epidemiology, antifungal susceptibility and virulence characteristics [1,26]. In vitro antifungal susceptibility testing has been conducted overseas, while there is still a lack of data regarding the antifungal susceptibility of clinical isolates in China. In this study, we systematically evaluated the activity of the three common echinocandins (including the novel rezafungin) through in vitro antifungal susceptibility testing of 1000 clinical *Candida* isolates and 150 *Aspergillus* isolates in comparison to fluconazole. Our results suggest that rezafungin has comparable activity to anidulafungin and caspofungin, and the echinocandins showed more potent activity than fluconazole against *Candida* species.

Azoles are a class of antifungals with a common pharmacological mechanism involving the inhibition of ergosterol synthesis in the fungal cell wall. However, azoles show the most common drug–drug interaction as inhibitors of cytochrome 450 enzymes, leading to a change in antifungal activity. For example, the antifungal spectrum of fluconazole against *Candida* species is relatively narrow, and the pharmacokinetics (PK) of voriconazole is non-linear [13,27]. It is necessary to monitor the drug concentration in azole therapy in the clinic to ensure its efficacy and safety. Echinocandins inhibit the synthesis of (1,3)-β-D-glucan and act on components that are specific to fungi rather than mammalian cells. With regard to their safety, dose adjustment of echinocandins is not necessary in patients with hepatic or renal insufficiency.

Echinocandins have a similar spectrum of in vitro antifungal activity, and there are few interactions between echinocandins and other drugs. Anidulafungin and rezafungin, which have been approved by the FDA, are still undergoing clinical trials in China. In our study, both anidulafungin and rezafungin were not inferior in activity when compared to the approved agent caspofungin, which is consistent with other published data [28,29]. The performance of the three tested echinocandins varied against different species, suggesting a different choice may be appropriate for different species, irrespective of other conditions. In the case of treatment failure and drug resistance, antifungal therapies are often applied long-term. Treatment should be continued even if clinical symptoms improve and patients are discharged during the course of treatment.

As a novel once-weekly echinocandin, rezafungin has great advantages for drug maintenance due to its long half-life of approximately 130 h, which allows more treatment options for patients and prolongs the drug administration cycle [30]. PK/PD (pharmacodynamics) studies show that plasma concentrations of rezafungin are high, which allows administration once a week in contrast to taking caspofungin or anidulafungin, which are taken once a day. Same as other echinocandins, rezafungin exhibits low toxicity due to its chemical and metabolic stability and solubility [31]. Rezafungin was recently approved by the FDA, in 2023, for the treatment of candidemia and IC in immunocompromised adults who have limited or no alternative options [19]. Data from the Phase 3 trial of rezafungin showed that it was non-inferior to caspofungin [17]. This suggests that local in vitro antifungal susceptibility testing would be effective in guiding clinical decisions related to the use of antifungals.

The echinocandin MIC values determined for all three echinocandins in our study are higher than for most previous similar surveillance studies, with a trend of two- to fourfold increase [29,32]. However, we evaluated the performance using the BMD method with the commercial Sensititre YeastOne (SYO) based on the MICs of anidulafungin against 20 *C. albicans* isolates. The essential agreement (EA, ±2 dilution) and categorical agreement were both ≥90% [33]. The QC values of every batch fell within the acceptable ranges. The sequencing results of the echinocandin-resistant *C. tropicalis* showed that all isolates displayed mutations on *FKS1*, which is consistent with the resistance rates interpreted by CBP of *C. tropicalis*. The results of several multicenter studies indicate that there is interlaboratory variability regarding the activity of echinocandins against *Candida* species [34,35]. Many factors can result in the interlaboratory variability of MICs, such as antifungal powder source, powder transportation condition, the type of microtiter plate, etc. [36]. It should be noted that we designed our study in accordance with the CLSI, and the resistance rates of *Candida* species was similar to those in previous research except in the case of *C. tropicalis.* We speculated that the reason for the higher MIC values is the possibility of adaptive evolution of the strains tested in our study, given that the tested strains were stored at −80 °C for 1–5 years.

We focused more attention on the analysis of drug-resistant *C. tropicalis* isolates in our study due to the increase in their incidence [6,37]. The resistance rates were much higher for *C. tropicalis* (about 8% to 40.67% in different antifungals) than the four other *Candida* species. Echinocandins showed an obviously better in vitro performance than fluconazole against *C. tropicalis*. The rates of susceptibility to rezafungin were similar to those for anidulafungin and caspofungin. As the rate of *C. tropicalis* echinocandin resistance was extremely high in our study, we searched for resistance mutations and found that all 13 echinocandin-resistant *C. tropicalis* isolates displayed alteration in *FKS1* S654P. We checked the clinical information of the isolates and found that 9/13 of them were isolated from the same neurology ward. The isolation date distribution of the nine echinocandin-resistant *C. tropicalis* strains seemed to be irregular. However, based on these results, we still recommend deep disinfection to be carried out in the ward and that more attention is devoted to environmental surveillance. Resistance to echinocandins was rarely reported in *Candida* species. However, the echinocandin susceptibility of *Candida* species has been reduced. A recent study in Japan reported 60.4% (32/53) of the *C. tropicalis* isolates intermediate to caspofungin [38]. The data of a 3-year study of *Candida* infections among patients with malignancy in Iran found that 17.6% *Candida* isolates were resistant to anidulafungin [39]. Prolonged drug exposure seems to lead to an alarming increase in resistance to echinocandins.

Rezafungin also shows potent activity against *A. fumigatus* and *A. flavus*, similarly to the results of previous studies [31,40]. In our study, the activity of rezafungin was shown to be comparable to that of caspofungin but lower than that of anidulafungin against *Aspergillus* species, although Pfaller et al. observed that caspofungin was less active than rezafungin and anidulafungin against all tested *Aspergillus* species [41]. A previous study reported that COVID-19 is one of the critical factors of IC and IA [42]. *Candida* and *Aspergillus* species detected in respiratory tract samples are commonly recognized as indicating colonization. Now, more attention should be paid to patients infected with novel coronavirus, especially in immunocompromised patients with recurrent candidaemia, out of concerns for the occurrence of COVID-19-associated candidemia and COVID-19-associated pulmonary aspergillosis [43,44]. Whether treatment is necessary for the isolation of *Candida* and *Aspergillus* species should be discussed taking into account the actual situation of the patients.

There are still some shortcomings of this study. We did not assess the in vitro activity of micafungin, which was also approved by the NMPA. We only chose the representative anidulafungin and most commonly used caspofungin in China for comparison. The shortage of micafungin data is the important limitation of our study. For personalized antifungal therapy, the three-dimensional interactions of fungi, drug and host must be integrated. More clinical trials are needed to collect in vivo clinical data for the use of the novel antifungals.

## 5. Conclusions

In vitro antifungal susceptibility testing can help in the clinical selection of effective antibiotics, based on consideration of pathogen sensitivity, toward reducing the treatment duration and economic costs for patients. However, not all laboratories are equipped to perform susceptibility testing. In our study, data on the in vitro activity of the antifungals against 1000 clinical *Candida* isolates and 150 clinical *Aspergillus* isolates allow providing recommendations for the clinical selection of antifungals. Rezafungin exhibited in vitro activity comparable to anidulafungin and caspofungin against the most common *Candida* and *Aspergillus* species. *C. glabrata* also showed higher rates of susceptibility to rezafungin. The potent activity of rezafungin is a basis for its potential clinical application. Advanced data are provided that support the use of rezafungin in China following its approval.

## Figures and Tables

**Table 1:** In vitro activity of anidulafungin, rezafungin, caspofungin and fluconazole against five common *Candida* isolates.

Species	Antifungal Agents	MIC Range (μg/mL)	MIC_50_ (μg/mL)	MIC_90_ (μg/mL)	GM (μg/mL)	Susceptibility (%)		
						S	I/SDD	R
*C. albicans* (n = 400)	ANI	≤0.016–1	0.06	0.12	0.068	99.25	0.25	0.5
	REZA	≤0.016–2	0.12	0.25	0.095	93.3		
	CAS	≤0.016–0.5	0.06	0.12	0.069	100		
	FLC	0.5–32	2	4	1.591	73.25	20	6.75
*C. glabrata* (n = 200)	ANI	0.03–1	0.125	0.25	0.140	84.5	13.5	2
	REZA	0.03–1	0.125	0.25	0.174	99		
	CAS	0.03–1	0.125	0.25	0.134	87.5	11	1.5
	FLC	0.5–128	2	4	1.613		98	2
*C. parapsilosis* (n = 200)	ANI	0.5–4	2	2	1.647	97.5	2.5	
	REZA	0.5–4	2	2	1.564	97.5		
	CAS	0.25–1	0.5	1	0.551	100		
	FLC	0.25–16	0.5	1	0.502	98.5	1	0.5
*C. tropicalis* (n = 150)	ANI	0.03–4	0.12	0.25	0.120	91.33		8.67
	REZA	0.03–4	0.12	0.25	0.163	90.67		
	CAS	0.03–8	0.06	0.25	0.110	90.67	0.67	8.67
	FLC	0.5–256	1	128	4.093	55.33	4	40.67
*C. krusei* (n = 50)	ANI	0.06–0.25	0.12	0.12	0.127	100		
	REZA	0.12–0.5	0.25	0.25	0.240	92		
	CAS	0.12–0.5	0.25	0.25	0.253	96	4	
	FLC	8–64	32	32	25.992			

ANI, anidulafungin; REZA, rezafungin; CAS, caspofungin; FLC, fluconazole; S, susceptible; I, intermediate; SDD, susceptible dose-dependent; R, resistant.

**Table 2:** In vitro activity of anidulafungin, rezafungin and caspofungin against fluconazole-sensitive *C. albicans* and fluconazole-intermediate/resistant *C. albicans*.

	Fluconazole-Sensitive *C. albicans*					Fluconazole-Intermediate/Resistant *C. albicans*				
	MIC_50_ (μg/mL)	MIC_90_ (μg/mL)	GM (μg/mL)	MIC Range (μg/mL)	Susceptibility (%)	MIC_50_ (μg/mL)	MIC_90_ (μg/mL)	GM (μg/mL)	MIC Range (μg/mL)	Susceptibility (%)
ANI	0.06	0.12	0.076	0.016 −0.25	100	0.06	0.25	0.067	0.016 −1	97.20
REZA	0.12	0.25	0.102	0.016 −0.5	94.54	0.06	0.35	0.09	0.016 −2	89.72
CAS	0.06	0.12	0.079	0.016 −0.25	100	0.06	0.12	0.072	0.016 −0.5	97.20

**Table 3:** In vitro activity of anidulafungin, rezafungin and caspofungin against two common *Aspergillus* species.

*Aspergillus* Species	Antifungal Drugs	MEC Range (μg/mL)	MEC_50_ (μg/mL)	MEC_90_ (μg/mL)	GM MEC (μg/mL)		
						WT	NWT
*A. fumigatus* (n = 100)	ANI	0.03–0.25	0.06	0.12	0.064		
	REZA	0.03–0.25	0.12	0.25	0.116		
	CAS	0.06–0.25	0.12	0.25	0.122	100	
*A. flavus* (n = 50)	ANI	0.03–0.12	0.06	0.12	0.059		
	REZA	0.03–0.25	0.12	0.12	0.110		
	CAS	0.03–0.25	0.12	0.25	0.142	50	

ANI, anidulafungin; REZA, rezafungin; CAS, caspofungin; WT, wild type; NWT, non-wild type.

**Table 4:** *FKS* alterations and MICs detected in strains of echinocandin-resistant *C. tropicalis*.

No.	MIC (μg/mL)				*FKS1* Mutation
	ANI	REZA	CAS	FLC	
CTR3	1	2	4	64	S654P
CTR8	2	2	4	128	S654P
CTR12	4	4	4	128	S654P
CTR16	2	2	4	128	S654P, R1220T
CTR17	2	2	4	128	S654P
CTR18	1	2	2	128	S654P, G324R
CTR20	1	2	2	64	S654P
CTR21	1	2	2	128	S654P
CTR22	1	2	2	128	S654P
CTR24	1	0.5	2	64	S654P
CTR25	1	1	2	64	S654P
CTR30	4	2	4	128	S654P
CTR36	1	2	4	128	S654P

## Data Availability

The original contributions presented in the study are included in the article/supplementary material, further inquiries can be directed to the corresponding authors.

## Supplementary Materials

The following supporting information can be downloaded at: https://www.mdpi.com/article/10.3390/jof10060397/s1, Table S1: Clinical characteristics of the 13 echinocandin-resistant clinical isolates.
