# Supplementary material for: In Vitro Activitiy of Rezafungin in Comparison with Anidulafungin and Caspofungin against Invasive Fungal Isolates (2017 to 2022) in China
Source: J Fungi (Basel). 2024 May 31;10(6):397. doi: 10.3390/jof10060397 (PMC11204387; doi:10.3390/jof10060397)
Supplement: Supplementary file 1 [file jof-10-00397-s001.zip › jof-2982965-supplementary.pdf]

Supplementary Table S1. Clinical characteristics of the 13 echinocandin-resistant clinical isolates.

NO.	ID	sex	age	date	source	hospital	ward
CTR3	RJX-0392	f	37	2018/12/22	puncutre fluid	RJ	ICU
CTR8	200396	m	64	2020/1/30	urine	DF	neurosurgery
CTR12	RJD24	m	62	2020/2/3	puncutre fluid	RJ	nephrology
CTR16	200486	m	47	2020/2/6	urine	DF	neurosurgery
CTR17	200778	f	36	2020/3/17	urine	DF	neurosurgery
CTR18	201047	m	63	2020/4/8	urine	DF	neurosurgery
CTR20	201252	m	60	2020/4/23	urine	DF	neurosurgery
CTR21	201363	m	68	2020/5/4	urine	DF	neurosurgery
CTR22	201564	f	55	2020/5/20	urine	DF	neurosurgery
CTR24	202548	m	67	2020/8/18	urine	DF	ICU
CTR25	203220	m	72	2020/10/19	urine	DF	SICU
CTR30	203302	m	66	2020/10/24	urine	DF	neurosurgery
CTR36	204245	f	52	2019/12/12	urine	DF	neurosurgery
